# Origin of the Genetic Code Is Found at the Transition between a Thioester World of Peptides and the Phosphoester World of Polynucleotides

**DOI:** 10.3390/life9030069

**Published:** 2019-08-22

**Authors:** Hyman Hartman, Temple F. Smith

**Affiliations:** 1 Earth, Atmosphere, and Planetary Science Department, Massachusetts Institute of Technology, Cambridge, MA 02139, USA; 2 BioMedical Engineering, Boston University, Boston, MA 02215, USA

**Keywords:** genetic code origin, tRNA accretion model, tRNA-synthetase, metabolism, thioester, nucleotidyltransferases, early peptides, ribosome

## Abstract

The early metabolism arising in a Thioester world gave rise to amino acids and their simple peptides. The catalytic activity of these early simple peptides became instrumental in the transition from Thioester World to a Phosphate World. This transition involved the appearances of sugar phosphates, nucleotides, and polynucleotides. The coupling of the amino acids and peptides to nucleotides and polynucleotides is the origin for the genetic code. Many of the key steps in this transition are seen in the catalytic cores of the nucleotidyltransferases, the class II tRNA synthetases (aaRSs) and the CCA adding enzyme. These catalytic cores are dominated by simple beta hairpin structures formed in the Thioester World. The code evolved from a proto-tRNA, a tetramer XCCA interacting with a proto-aminoacyl-tRNA synthetase (aaRS) activating Glycine and Proline. The initial expanded code is found in the acceptor arm of the tRNA, the operational code. It is the coevolution of the tRNA with the aaRSs that is at the heart of the origin and evolution of the genetic code. There is also a close relationship between the accretion models of the evolving tRNA and that of the ribosome.

## 1. Introduction

There are two competing theories for the origin of life that are based on Darwinian selection; the RNA World and the Clay World. They both assume a replicating and a mutating entity that has catalytic capacity. The selection in both cases is on the evolution of the catalytic capacities whose products increase the replication and survivability of in the first case, of the RNA world, the replicating RNA and its catalytic capability and in the second case, of the Clay world, the replicating Clay and its catalytic capability [1].

The genetic code and the translation system had to have arisen in an environment containing, at a minimum, the molecular building blocks (e.g., amino acids and nucleotides). “A general scheme is proposed, involving the fixation of CO_2_ and N_2_, that led to the evolution of intermediary metabolism. The result is the evolution of a complex system from a simple one. Following the logic that core metabolism recapitulates biopoiesis, we begin by describing, an autotrophic origin of metabolism based on self-replicating iron-rich clays, transition-state metals, disulfide and dithiols, and UV radiation” [2].

A scenario for the origin of life consistent with the origin and evolution of metabolism started at the surface of an early outgassing Earth. In the surface hot springs, the water was rich in ferrous ions, magnesium ions, and silicate ions that formed iron-rich clays. The waters of the hot spring also included transition-state metal ions (i.e., Mn, Cu, Zn), adding to the catalytic capabilities of the iron-rich clays. These self-replicating clays would photochemically fix CO_2_ into organic acids and gradually evolve into the sulfide-rich region acquiring N_2_ fixation in the process.

A Thioester World had come into being. The entry of phosphate into the evolving catalytic network expanded the metabolic network. The Phosphate World had come into being [3,4].

The onion heuristic view claims that a complex system like a metabolic network started simple and grew more complex by layers being added, as for example, the Thioester World evolving into the Phosphate World by adding a new layer dominated by phosphate. Looking at intermediary metabolism, in particular anabolism, one is immediately struck by the central role played by the (reverse) citric acid cycle. If we apply the method of the onion heuristic view, it can be conjectured that the citric acid cycle came first and was followed by the amino acids, lipids, nucleotides, and carbohydrates.

## 2. Metabolism

### The Evolution of Metabolism Can Be Considered to Have Undergone Four Distinct Phases

**The first phase began with Iron-rich clays with inputs (CO_2_, H_2_O).** The formation of dicarboxylic acids such as oxalic acid and glyoxylic acid are synthesized from CO_2_ and H_2_O by iron-rich clays and light which is the source of energy. These dicarboxylic acids are catalysts in the formation of iron-rich clays themselves. The iron-rich clays in the light are the center from which metabolism started [3].

**The second phase brought in sulfur; Iron-rich clays with inputs (CO_2_ H_2_O H_2_S, Fe_2_S_2_, and Fe_4_S_4)_.** The entry of sulfur brings in the thioesters and the Fe_2_S_2_ and Fe_4_S_4_. This results in the reverse citric acid cycle and the polymerization of acetyl-thioesters, resulting in the biosynthesis of the fatty acids. This step is continuous with the next step, which is the entry of nitrogen.

**The third phase brought the addition of nitrogen and created the “Thioester World”: Iron-rich clays with inputs (CO_2_, H_2_O, H_2_S, Fe_2_S_2_, Fe_4_S_4_, and N_2_ )**. Fixation of nitrogen occurred here on the surface of an iron sulfide [4,5]. The amino acids were synthesized from the reverse citric acid cycle giving rise to the metabolic metric —the number of catalytic steps in the biosynthesis of 16 amino acids from that cycle. The four amino acids Phenylalanine, Tyrosine, Tryptophan, and Histidine are not derived from the reverse citric acid. The aromatic amino acids may have been synthesized by Fe-serpentines and Iron saponites [6]. The synthesis of di, tri and tetrapeptides had been formed from their amino acid thioesters [3,4]. These initial peptides and their combinations had been able to provide additional catalytic activities to those of the iron-rich clay surfaces.

**The fourth phase brings in Phosphate: Iron clays with inputs (CO_2_, H_2_O, H_2_S, Fe_2_S_2_ Fe_4_S_4_, N_2_ and N_2_PO_4_**) and the biosynthesis of nucleotides. The formation of pyro-phosphate is followed by the biosynthesis of the sugar phosphates such as 5-phosphoribosyl-1-pyrophosphate (PRPP) the phosphate sugar that initiates the biosynthesis of purine nucleotides.

**Figure 1:**
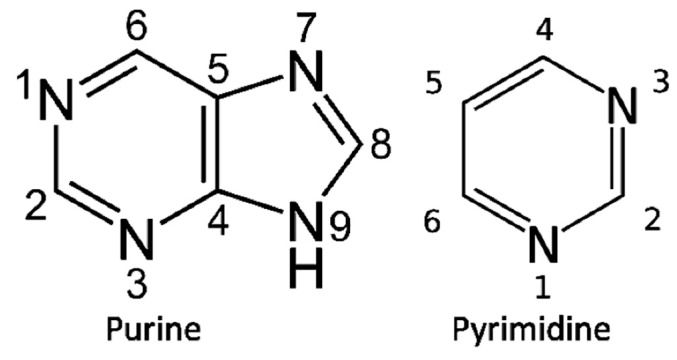
The basic Purine and Pyrimidine bases. The numbering is used in the text to identify the sources of their atoms in biosynthesis.

The biosynthesis of purine bases (Figure 1) begins with phosphoribosyl-1-pyrophosphate (PRPP) the phosphate sugar that initiates the biosynthesis of Purine nucleotides. The biosynthesis of the Purine base involves the glycine molecule (atoms 4, 5, 7), the amino nitrogen of aspartate (atom 1), amide nitrogen of glutamine (atoms 3, 9), components of the folate-one-carbon pool (atoms 2, 8), and carbon dioxide. In the biosynthesis, IMP ((Inosine monophosphate) is the first nucleotide formed. It is then converted to either AMP (adenosine monophosphate) or GMP (guanosine monophosphate). The biosynthesis of Histidine is closely related to Purine biosynthesis.

The pyrimidine biosynthesis begins with carbamoyl phosphate (atoms 2, 3) that condenses with aspartate (atoms 4, 5, 6, 1), resulting in dihydro-orotate which is then added to PRPP to form the nucleotide. Further steps are needed to form Uridylic acid and Cytidylic acid. The Nucleotides Guanylic acid, Cytidylic acid, Adenylic acid, and Uridylic acid are then polymerized by the proto-CCA nucleotidylsynthetases formed in the Thioester World.

## 3. Genetic Code 

The search for the origin of the genetic code began by examining the extant genetic code, which is a mapping of the sixty-four codons (triplet nucleotide sequences), to the twenty amino acids and three termination codons, (Table 1). The genetic code uses a set of four nucleotides normally displayed in a single letter code: A for Adenylic acid, G for Guanylic acid, C for Cytidylic acid, and U for Uridylic acid. There are twenty amino acids encoded by 64 codons and presented in a four-by-four table containing the three letter abbreviations for each of the twenty common amino acids, as shown in Table 1.

A structural relationship between the general classes of amino acids and the middle nucleotide of the codon has been well recognized. For example, the middle nucleotide U codes for hydrophobic amino acids, and the middle nucleotide A codes for hydrophilic and the larger neutral amino acids. The middle nucleotide C codes for small neutral amino acids. On the other hand, the middle nucleotide G points to a unique case of Arg as a large and an unlikely amino acid sharing a codon with serine. This uniqueness is eliminated if a smaller positive amino acid is substituted as a biochemical precursor for Arginine.

### 3.1. The Evolution of the Genetic Code

**Figure 2:**
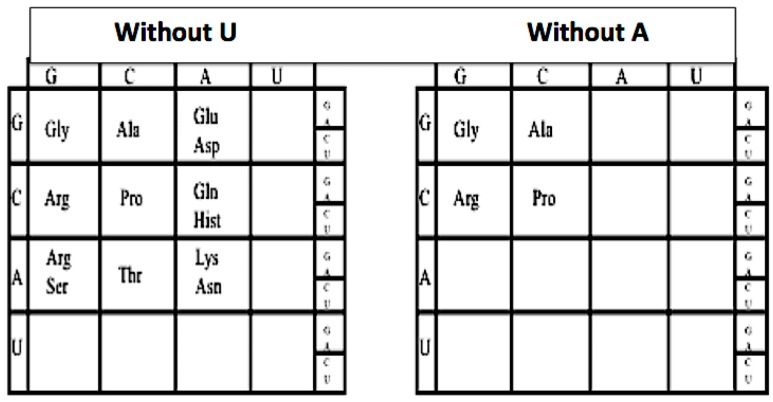
The reduced nucleotide genetic coding tables.

This can be seen by sequentially omitting the columns headed by U and A, and the rows headed by U and A, one can peel back the genetic code to its earlier form, see Figure 2. For example, if we omit the U column and row from the genetic code: The hydrophobic amino acids Val, Leu, Ile, Met, and Phe are omitted as are Tyr, Trp, and Cys, as well as the three termination codons UAA, UAG, UGG, leaving a GCA code. Next by omitting the A column which then omits Glu/Asp, Gln/Hist, and Lys/Asn. This removes the two negative amino acids Glu and Asp and their neutral versions Gln and Asn and two positive amino acids Lys and Hist.

The codons in the A column are split due to the wobble in the third base. Omitting the A row: removes Thr, and the split codons for Arg/Ser. This leaves a simple GC code for Gly, Pro, Ala, and Arg. This GC form of the first code was proposed in 1975 [7]. Reversing the sequence implies that the genetic code evolved in an onion-like fashion, beginning as simple GC code and growing by adding layers (e.g., by adding A and forming the GCA code and finally adding U and forming the GCAU code).

**Figure 3:**
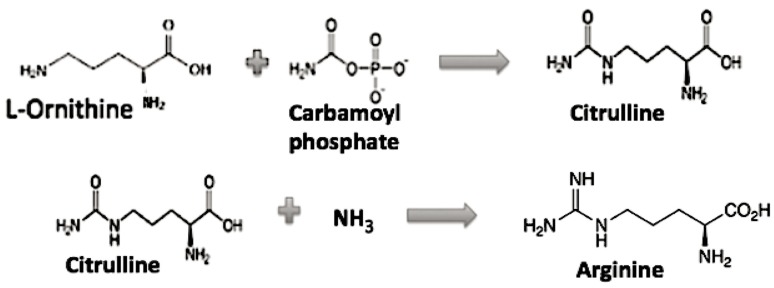
The Ornithine to Arginine pathway.

The role of Arginine in the evolution of the genetic code appears to need additional discussion, as it is the only one left in the GC code which is activated by a different tRNA synthetase class and requires multiple biosynthetic steps. If Arg is then not considered to be among the early available amino acids, then what might the CGX codons have coded for early on? First, note Arg is related biochemically to a simpler amino acid ornithine as its precursor in its biosynthesis, see Figure 3 [8].

Why then was Ornithine chosen rather than Lysine as a precursor to Arginine? The answer is that the difference in chain length between Ornithine and Lysine is related to the formation of alpha helices. It was shown experimentally that the helix propensities for the basic amino acids increase with the length of the side-chain in the rank order 2,3-Diamino-L-propionic acids < 2,4-Diamino-L-butyric acid < 2,5-Diaminopentanoic acid (ornithine)< 2,6-Diaminohexanoic acid (Lysine) [9]. The number of side-chain atoms beyond the carboxyl group of the amino acid ending in the NH2 group is the length of the carbon chain in this family of amino acids that determines whether a polypeptide alpha helix will form.

In a GC code coding for Glycine, Proline, Alanine, and 2,3-Diamino-L-propionic acid (Diapr), the earliest coded peptides were not alpha helical, rather mainly simple random coils and beta hairpins as proposed by Soding and Lupas [10]. If one substitutes 2,3 Diamino-L-proprionic acid (Diapr) in place of Arginine (or Ornithine), then there would be a simple chemical relationship among three of the G column amino acids, Diapr, Serine, and Cystine. Diapr has an NH2 group while Cystine has an SH in place of the OH of Serine. This gives us a similarity in their structure and perhaps in their early biosynthesis. As the genetic code evolved to the GCA code (AAA, AAG coding for Lysine) and to the GCAU (hydrophobic amino acids), then the later peptides now incorporating Lysine rather than Diapr were able to form alpha helices. Arginine resulted when an Ornithine reacted with carbamoyl phosphate that resulted in a guanidino group.

We now have a conjecture for the origin and evolution of the genetic code (GC code --> GCA code ---> GCAU code) The evidence for this conjecture is to be found in the translational apparatus of the cell in which this code is expressed. The obvious place to start is in the set of aminoacyl-tRNA synthetases.

### 3.2. Aminoacyl-Transfer RNA Synthetases Contain the Genetic Code

It is in the aminoacyl-tRNA synthetases (aaRS) where each particular amino acid is activated and attached to its cognate tRNA. The majority of synthetases are composed of three domains: The catalytic domain where the amino acids are activated and passed on to tRNA or a thiol; the anticodon domain, which recognizes the anticodon stem loop of the extant tRNA; and the editing domain, which removes a mischarged amino acid from its bound tRNA. The catalytic domain [11] of the aminoacyl-tRNA synthetases (aaRS) is considered to be the original encoding module of the aaRS while the anticodon domain and the editing domains are considered to be later evolutionary additions to the catalytic domain. The reactions carried out by the catalytic domain are:

1. Amino Acid + ATP → Aminoacyl-AMP + PPi
2. Aminoacyl-AMP + tRNA → Aminoacyl-tRNA + AMP

There are two classes of these synthetases, which are characterized by their different catalytic domain structures. Class I’s catalytic domain has a Rossman fold and activates Val, Leu, Ile, Met, Cys, Tyr, Trp, Glu, Gln, and Arg, while class II’s catalytic domain is unique and has an antiparallel beta sheet backed by an alpha helix. Class II synthetases activate Gly, Pro, Ala, Thr, Ser, Hist, Asp, Asn, Lys, and Phe. The Class II synthetases are considered to be older than the Class I synthetases. This is based on the fact that the majority of the hydrophobic amino acids are activated by class I synthetases and were unlikely to have been available for early polymerization, while Glycine, Alanine, Proline, and Aspartic acid were available. The evidence for this is based on the metabolic metric, which orders the entry of the amino acids into the genetic code by counting the number of catalytic steps involved in the biosynthesis of the amino acid from the reverse citric acid cycle [12]. It is in the catalytic domain of the class II synthetases and its interaction with the XCCA of the proto-tRNA that evidence for the first GC code arises.

### 3.3. The Catalytic Domain of the Class II Aminoacyl-tRNA Synthetases

**Figure 4:**
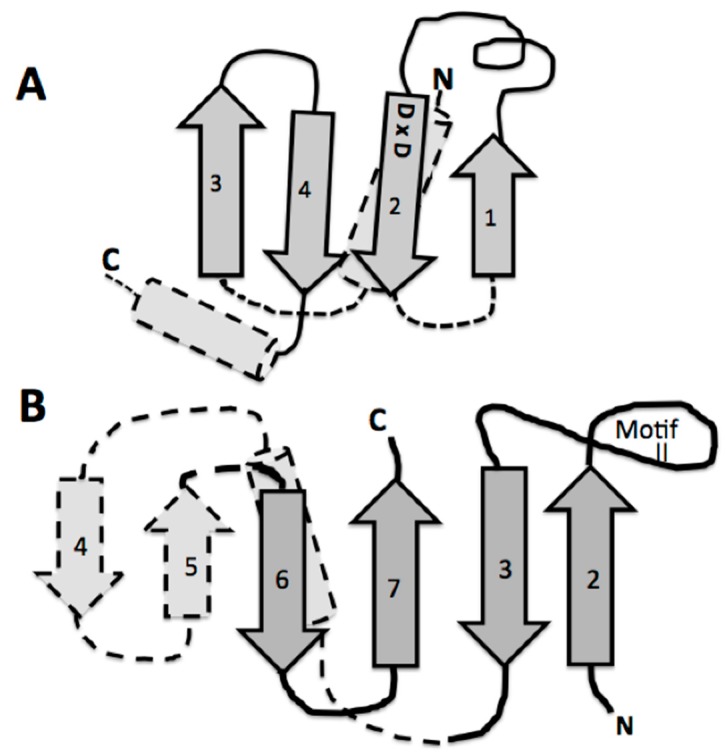
Comparison of the similarity of the double beta hairpin cores of (**A**) the bacterial CCA adding enzyme head domain and (**B**) the class II tRNA synthetase minimum core of the catalytic domain. The minimum catalytic domains are in dark gray. Note the beta hairpin components 3 and 2 in (**B**), which define the motif II loop; and 1 and 2 in (**A**), which contain the two Aspartic acids. The strand numbering for (**B**) is as in reference [11] for the entire traditionally-defined class II catalytic domain.

A detailed look at the catalytic domain of these assumed first tRNA synthetases is warranted. This catalytic core is composed primarily of an antiparallel beta sheet constructed from two or three beta hairpin segments [11]. In the extant structures, two of these beta hairpin substructures are connected by an alpha helix, which is on the underside of the active site pocket formed by only two beta hairpins, one hairpin with a large end loop (see Figure 4B). It is this large end loop that forms a lid over the active site pocket and that is evolved into the defining motif II of the class II aminoacyl tRNA synthetase. This motif II loop and the four beta strands in dark gray in Figure 4B, compose the full binding site for the ATP, the amino acid to be activated, and the CCA-3′ acceptor end of the tRNA. This structure was proposed as the proto-class II synthetase [11].

If the origin of the genetic code is in the class II synthetase catalytic core, then assuming that core existed before the fully functioning code, a problem presents itself: Where did the peptides forming these beta hairpins come from? The answer proposed here, is in the Thioester World. There the polymerization of amino acid Thioesters generated short peptides, some of which formed catalytic active beta hairpins [13].

### 3.4. The Thioester World and Aminoacyl-tRNA Synthetases

De Duve proposed an early proto-metabolism of amino acids and peptides in a Thioester environment, a world in which there were no Phosphates and thus no nucleotides [14]. He went further and proposed “that clues to the nature of that early proto-metabolism exists within modern metabolism” [15]. In collaboration with the Segre group at Boston University [4] such a proto-network was identified within the modern metabolic network (The Kegg metabolic network). A proto-metabolic network based on Thioesters was obtained when Phosphate was omitted from this Kegg metabolic network. This proto-metabolism had been capable of synthesizing amino acids and fatty acids. The network displays hallmarks of prebiotic chemistry (e.g., iron sulfur cofactors) [4]. The existence of a Thioester environment prior to translation points to the existence of metabolism that preceded the existence of translation. Unlike the phosphate esters which are involved in the activation of amino acids to form peptides, the core metabolism of the Thioester World provides amino acid thioesters allowing for their polymerization to form peptides. In the Thioester World, the metabolic metric can be defined as the number of steps from the reverse citric acid cycle to form an amino acid. The amino acids Glycine, (GG *) Proline (CC *), and Alanine (CG *) are among the earliest available amino acids as based on this metabolic metric. There is a close relationship between core metabolism and the Thioester world, especially in the case of the synthesis of 16 of the 20 amino acids. The exceptions are Histidine, Phenylalanine, Tyrosine, and Tryptophan [12].

Jakubowski has studied the thioester formation by class II Aminoacyl-tRNA synthetases and he summarized these findings as follows, ‘‘These and other data support a hypothesis that the present-day Aminoacyl-tRNA synthetases had originated from ancestral forms that were involved in non-coded Thioester-dependent peptide synthesis, functionally similar to the present-day non-ribosomal peptide synthesis by multi-enzyme thiol-template systems” [16]. This finding is also consistent with a recent example of an atypical Seryl-tRNA synthetase found in a methanogenic Archaea, which lacks a tRNA binding site and instead transfers the activated amino acid (Aminoacyl-AMP) to a sulfhydryl group found on the phosphopantetheine (related to Coenzyme A) which is bound to a carrier protein forming a Thioester. The activity of this atypical Seryl-tRNA synthetase is ‘‘reminiscent” of the adenylation domains in non-ribosomal peptide synthesis involving a Thioester [16,17]. Thus, before the development of mRNA-coded protein synthesis, ancestral aaRSs facilitated formation of Aminoacyl-Thioesters. Then this early catalytic proto-class II synthetase core, capable of both Thioester and Phosphate ester activation of amino acids, can be considered the link in the transition between a Thioester World and a Phosphate ester World [8].

From such amino acid thioesters, peptide formation is certainly possible. Note that peptide hairpins, such as those forming the catalytic core of the class II synthetases, are considered to have been among the earliest peptide structures [10] and can be formed by the polymerization of amino acid thioesters. The beta hairpins are known to be involved in many other catalytic activities [13]. As noted below such catalytic beta hairpins form a significant part of the core of other key early translational system proteins, such as the CCA nucleotidyltransferases and tRNA ligases. Both of these protein catalytic activities are essential for understanding the coupling of the synthetase’s continuing coevolution with that of the tRNA.

## 4. Origin of the tRNA

The first proto-tRNA is proposed to have been a short three or four polynucleotide, for example, 3′-XCCA-5′ [18,19], that interacted with the catalytic core of a proto-class II aminoacyl-tRNA synthetase (aaRS) pictured in Figure 4B. First why CCA? The required base stacking in the extant class II synthetases suggests that the Adenine is essential in base stacking to coordinate the positioning of the incoming ATP and AMP of the activated the amino acid. The other two bases, the Cytosines, thus must not be Adenine.

The likely origin of this proto-tRNA is proposed to involve a CCA-like nucleotidyltransferase beta structure. There are two distinct extant CCA adding enzymes, one in Archaea and a second in Bacteria. Both CCA enzymes are composed of four domains, which are labeled head, neck, body, and tail. The head or catalytic domain in both of these enzymes are homologous structures, whereas the neck, body, and tail are not. Of interest here, is this common invariant catalytic core in the Head. In the extant CCA enzyme, the catalytic Head behaves like the glycosyltransferases in their mechanism of transferring an activated sugar phosphate in the ligation step. There is a β-DxD-β metal-binding motif in the active center of glycosyltransferases and nucleotidyltransferases [19]. An examination of the CCA catalytic head DxD peptide (Aspartate x Aspartate) structure shows an interesting similarity to that of the class II synthetase catalytic core (Figure 4). Both are constructed from beta hairpins, see Figure 4. Part of this interest here is that both of these enzymes are nucleotidyltransferases, in that they both transfer nucleotides to recipient molecules. In the case of the CCA enzyme the recipient molecule is a 3′ nucleotide of the tRNA, while in the case of the class II aminoacyl-tRNA synthetase, it is to an amino acid forming the Aminoacyl-AMP, which is then transferred to the CCA of the tRNA.

The full catalytic pocket of the modern CCA enzymes is formed by the interaction between the catalytic core, the Head, and an alpha helical domain in the Neck [20]. As these enzymes do not use a polynucleotide template, base specificity is in the neck where amino acids form Watson/Crick-like hydrogen bonds with just the nucleotide base. Now, assuming the earliest proto-transferase was not this complex, the specificity must have come from some other peptides or even a short polynucleotide generated by the similar kinds of enzymes.

The recent determined structure of a tRNA ligase from a T4 phage has in its N-terminal domain a set of beta structures that dominate the catalytic core [21]. This core of an extant tRNA ligase is composed of two beta hairpin substructures backed by a helix or two, almost as if composed of two CCA-like catalytic cores. There is even a functional similarity between the aaRS and the extant tRNA ligase nucleotidyltransferase. Both form an aminoacyl-AMP intermediate: The tRNA ligase forms a Lysyl-AMP intermediate with the AMP that is subsequently added to the 5′ phosphate end of a polynucleotide RNA. This is then followed by a third step when the AMP is removed upon the ligation with the 3′ OH of a second polynucleotide RNA. The independent functioning of the nucleotidyltransferase activity of these ligases has been demonstrated by mutational studies [22]. It is clear that the modern tRNA ligases have undergone extensive evolution, yet the proto-ligase could have been carried out by two primitive proto-CCA enzyme-like entities. Again, the catalytic cores of the tRNA ligases, the nucleotidyltransferases (e.g., CCA enzyme), and the class II aminoacyl-tRNA synthetases are all built from beta hairpins, supporting the idea that their proto forms were formed in the Thioester world [14,15].

### 4.1. The Accretion Model of the tRNA

**Figure 5:**
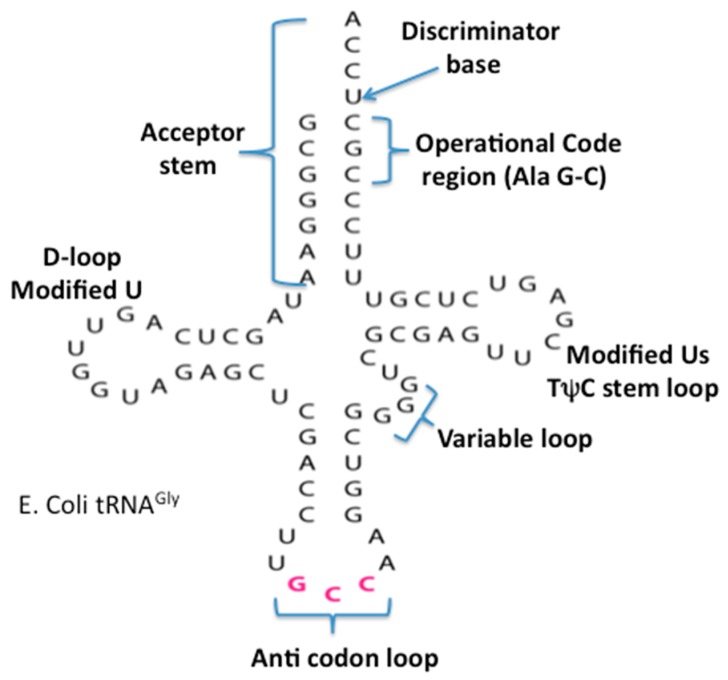
The secondary cloverleaf structure of *E. coli’s* tRNA Gly. The discriminator base, the operational code region, and the various stems and loops are labeled in their traditional manner.

The origin and evolution of tRNA is based on the proposed appearance of the proto-nucleotidyltransferase catalytic cores arising in the Thioester that generated nucleotide dimers, trimers and tetramers as well as the trimer CCA. In addition, the ligation of these would have formed single-stranded short RNAs. These coupled with hybridization formed double stranded helices. The modern tRNA structure suggests an assembly from such short helices. For example the extant secondary structure of a typical extant tRNA, shown in Figure 5, can be dissected into five segments or domains: The XCCA attached at the 3′ end of the tRNA; the acceptor arm, which has a stem of seven base pairs; the Anticodon stem loop, which has a stem of five base pairs; and the anticodon loop containing seven unpaired nucleotides. Three of these unpaired nucleotides form the anticodon: the TΨC stem loop, which has a stem of five base pairs, and a loop containing seven unpaired nucleotides in which CGA are isomorphic with the three anticodon bases in the anticodon stem loop; the D stem loop, which has a stem of four or five base pairs and a loop containing seven or eight unpaired nucleotides, with an invariant triplet, GGU, in the loop. Note that the D and T loop names reflect various nucleotide modifications found in the majority of extant tRNAs.

### 4.2. The Operational Code

The initial expansion of the acceptor arm from the XCCA and its relationship to the catalytic domain of the class II synthetases is reflected in the discovery by Hou and Schimmel [23], that a major determinant in the specificity of the class II-tRNA synthetase coding for Alanine was in the G–U pair (3–70) and was found in the acceptor arm. De Duve went on to describe: “A second genetic code still largely un-deciphered is written into the structure of aminoacyl-transfer RNA synthetases” [24].

Schimmel noted: “Because the amino acid-trinucleotide algorithm of the genetic code is established by the specific aminoacylations of tRNAs, the sequences and structure of tRNAs have long been investigated with the idea of learning about the possible origin of the code. The idea is that elements of the early code might [yet] appear in parts of the tRNA structure other than the anticodon. These parts might represent primordial components of the tRNA molecule, which possibly served as structures associated with the aminoacylations of particular amino acids” [25]. He was reporting on a finding by Rodin and Ohno [26] of codons and anticodons in the arms of the tRNA.

Rodin and Ohno, by studying the acceptor arms of numerous tRNAs, identified an expanded view of this code, now called the operational code. They found “In contrast with anticodons, which are built from all four nucleotides, their double-stranded precursors in the 1-2-3 positions of acceptor arms appear as doublets or triplets almost invariably composed of G-C and C-G base pairs. Even in many modern synthetases, including the deep Archaea, specific amino-acylation of the acceptor terminus operates within the G-C variety of first three acceptor base pairs” [26]. It should be pointed out that as early as 1975 it was hypothesized that the first genetic code was a GC code for Gly, Pro, Ala and a positively charged amino acid [7].

Rodin and Ohno proposed [27] a detailed synthesis of the acceptor arm from the trimers 5′-GGC-3′ and 5′-GCC-3′ and the tetramers 5′-ACCA-3′ and 5′-UGGU-3′. Resulting in the proposed initial acceptor arm:

3′-A-C-C-A-C-C-G-U-G-G-U-5′
      5’-G-G-C-A-C-C-A-3’.

Here then is a proposed origin of the GC operational code in the tRNA acceptor arm.

**Figure 6:**
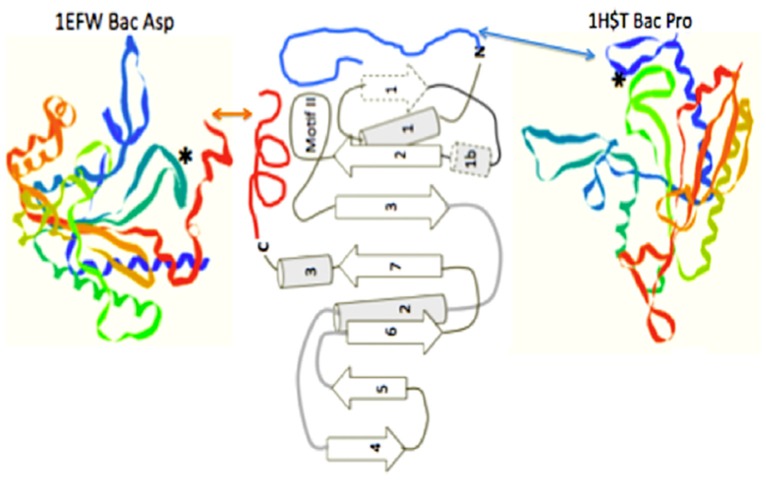
The class II synthetase catalytic core showing two alternate operational code-contacting peptide extensions, one on the N-terminal, as seen in the bacterial Proline aaRS structure 1HST.pdb, and one on the C-terminal, as seen in the bacterial Aspartic acid aaRS structure, 1EFW.pdb. Figure is adapted from [11].

In order to access the information in this initial form of the operational code, the catalytic domains of the class II synthetases had to be able to “read” the upper acceptor stem of the primordial tRNA. The minimum catalytic core of the assumed early class II synthetases does not make significant contact with the arm of the tRNA. These minimal enzymes have added relatively short peptides to create N- or C-terminal extensions, often as alpha helices, to their minimal catalytic cores. It is these extensions, which make the explicit contact with the operational code region of the extant t-RNAs [11], (see Figure 6). This is an excellent example of the co-evolution of the proto-tRNA and the proto-class II synthetases in generating the amino acid to RNA coding association of the Archaic GC operational code in the tRNA arm.

The next step in the evolution of the proto-tRNA is the formation of the stem loops. Schimmel and his group [28] tested the operational code with a variety of truncated arms of the tRNA. These truncated tRNAs were stem helices of various length with the XCCA at the 3′ end. These were suggested as possible precursors of the expanding proto-tRNA and as well as the anticodon stem-loop structure of the tRNAs. One of these truncated arms had an overhang of a single-stranded 5-mer at the five prime end of the second strand, yet was still properly charged [28]. This overhanging stem is a reasonable initial model for the synthesis of an anticodon stem loop. The upper triplets of this stem helix contained the archaic GC code (operational code) [28]. In a manner similar to the one found in the upper triplets of the extant tRNA arm, a 5-mer containing a trimer based on GC plus a dimer provided the GC code in the stem helix and also in the overhang at the 5′ end. The resulting structure was closed by ligating another dimer to form a seven-nucleotide closed loop with the operational code not only in the stem but also in the related anticodon in the loop. The GC code was in the helix and in the loop. This was the first set of stem loops with the GC code in the loop. The CGA triplet in the loop of the T stem loop and the GGU triplet in the loop of the D stem loop are remnants of a GC code. The later anticodon stem loops incorporated the anticodons for the GCA code and then for the GCAU code [29,30,31].

### 4.3. The Maturation of tRNAs

**Figure 7:**
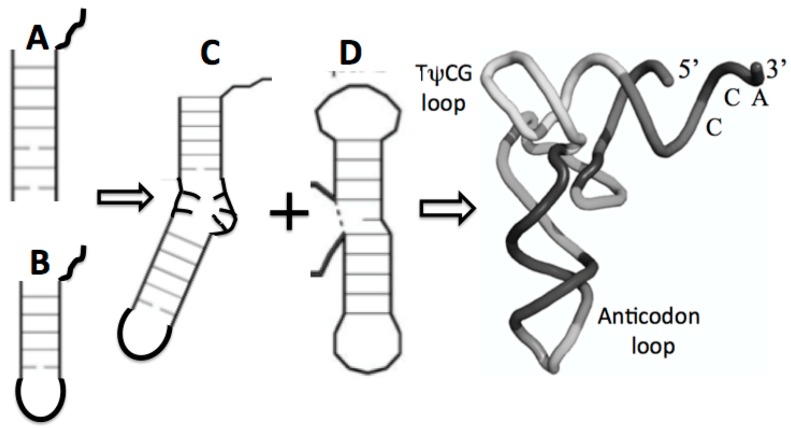
Possible maturation of the full tRNA: (**A**) arm with CCA attachment, fused with (**B**) stem loop with CCA attachment resulting in (**C**) the boomerang that then fused with (**D**) the dumbell resulting in the tRNA cloverleaf secondary structure in Figure 5 and the full 3D, folded tRNA structure pictured here.

There are two proto-tRNA-type structures available, an arm and stem loop, each containing the CCA end. The ligation of two such sub structures has been referred to as the boomerang, (Figure 7C). A second ligation of two stem-loop substructures formed the so-called dumbbell, (Figure 7D). Such a boomerang functioning as a truncated tRNA has been found in the mitochondria of the worm Enoplea. “There, the mitochondria of some metazoans, contain deviations from the consensus tRNA structure, some tRNAs lack both the D- or T-arm without losing their function. In Enoplea, this miniaturization comes to an extreme, and functional mitochondrial tRNAs can lack both arms, leading to a considerable size reduction, and a flexible link between the anti-codon arm and the CCA acceptor arm. Hence, it is believed that the single connector elements in these transcripts have an increased flexibility in order to allow the formation of an extended 3D tRNA shape, bringing the anticodon loop and 3′-terminus into a conserved distance that is required for acceptance by the ribosome and, consequently, participation in translation. This shape deviates from the classical L form and is more boomerang-like [32].

The ligation between the stem loop (Figure 7B) and the anticodon stem loop (Figure 7B) containing the anticodons generated a second structure referred to as the dumbbell, as shown in Figure 7D. Finally, the ligation of these two substructures, the boomerang and the dumbbell, resulted in the secondary structure of the tRNA, the cloverleaf, as shown in Figure 5.

The 2D cloverleaf representation of the tRNA in Figure 5, when compared to the 3D structure in Figure 7, shows a complex change in its configuration due to the coevolution of the proto-tRNA with the aminoacyl-tRNA synthetases and the large subunit of the ribosome (**LSU**) and the small subunit (SSU) of the ribosome. The accretion model of the tRNA was implicit in forming the seed for the accretion models of the LSU and the SSU of the ribosome [33].

### 4.4. The Accretion Models of the tRNA and the Seeds for the Ribosome

The proto-tRNA and its interactions with the proto-ribosome points us to the accretion model for the ribosome as proposed by Petrov et al. [33]: “In phase 1, ancestral RNAs form stem loops and minihelices. In phase 2, the LSU catalyzes the condensation of nonspecific oligomers. The SSU (small subunit of the ribosome) may have a single-stranded RNA-binding function. In phase 3, the subunits associate, mediated by the expansion of tRNA from a minihelix to the modern L shape. LSU and SSU evolution is independent and uncorrelated during Phases 1–3”.

In phase 1, the start of the accretion model for the LSU and the SSU began with a set of ancestral RNAs. The source for this set of RNAs was not identified. It is here proposed that the source for their accretion model [33] was the same as that for the accretion of the tRNAs, which began with di-, tri-, and tetra-nucleotides ligated to form various additional stem-loop structures that supplied the seeds for the accretion model of the LSU and SSU of the Ribosome. An example was the minihelix formed by ligation of a seven-member helix (arm), with the GC code in the arm, and five-member stem, with a seven-member loop containing a CGA triplet. The difference between the boomerang and the minihelix is the ligation took place without the XCCA on the arm and the TΨCG stem loop.

**Figure 8:**
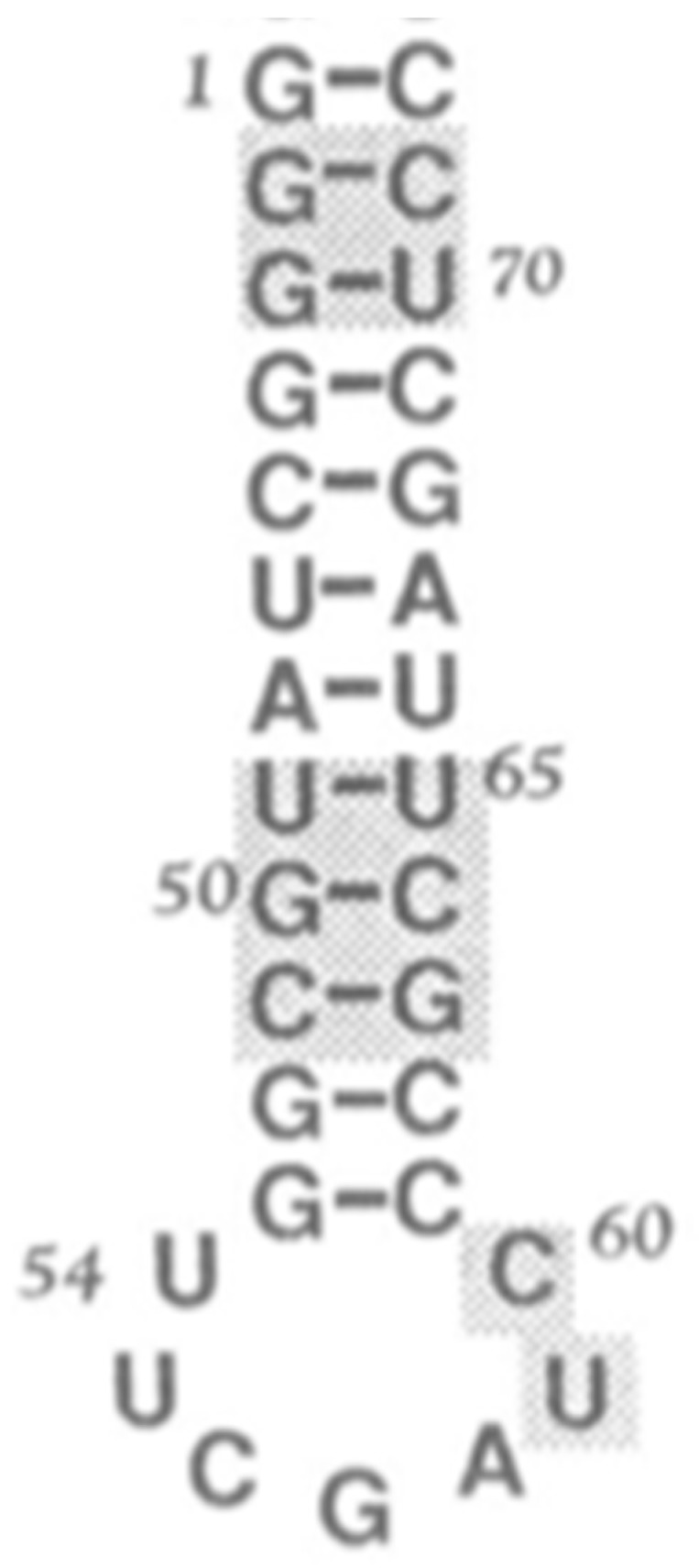
Minihelix example, proposed by Tamura [34] as a seed for the ribosomal subunits.

The seed for the large subunit of the Ribosome was the minihelix, similar to those forming the proto-tRNAs, Figure 8. A number of suggestions have been made for the formation of the PTC of the LSU. However, the one by Tamura is most relevant as it involves the tRNA. Tamura [34] proposed that the minihelix was an ancestral precursor of both the modern tRNA and of the large ribosomal subunit PTC (peptidyl transferase center). He claimed that “...considering the RNA minihelix as the molecule of origin can definitely lead us to attaining our goal of elucidating the emergence of the modern peptide synthesis machinery of the ribosome”. This idea was examined by Farias et al. [35] by reconstructing probable ancestral tRNA sequences from extant tRNAs and comparing them with the large subunit PTC RNA from a range of different organisms. The result showed a remarkable sequence identity.

Note, a recent model derives the tRNA from three minihelices similar to those proposed for the accretion models of both the ribosomal subunits. “The conserved archaeal tRNA core (75-nt) is posited to have evolved from ligation of three proto-tRNA minihelices (31-nt) and two-symmetrical nine-nt deletions within joined acceptor stems (93 − 18 = 75-nt)” [36].

### 4.5. Early Function of the LSU Proteins

What was the initial function of the proto-LSU? It was examined by Schimmel et al. [31] in testing the operational code with a variety of truncated tRNAs with XCCA at the 3′ ends. The truncated tRNAs were able to interact with the catalytic domain of the class II aminoacyl-tRNA synthetase and add the amino acid to the CCA. However, if the arm was long enough it bound to the PTC of the LSU and transferred the amino acid to a growing peptide. Thus, the most likely early function of the proto-LSU, composed of little more than the PTC, was to have bound two minihelices; and by bringing the activated amino acids attached to the XCCA into contact allowed the formation of dipeptides. It generated longer peptides that would depend on the further formation of the tunnel. There was a GC code (operational code) in the arm of the proto-tRNA that interacted with class II synthetases catalytic modules and perhaps the GC code in the arm stabilized the formation of the dipeptide with the PTC of the proto-LSU.

### 4.6. The Origin of the SSU

**Figure 9:**
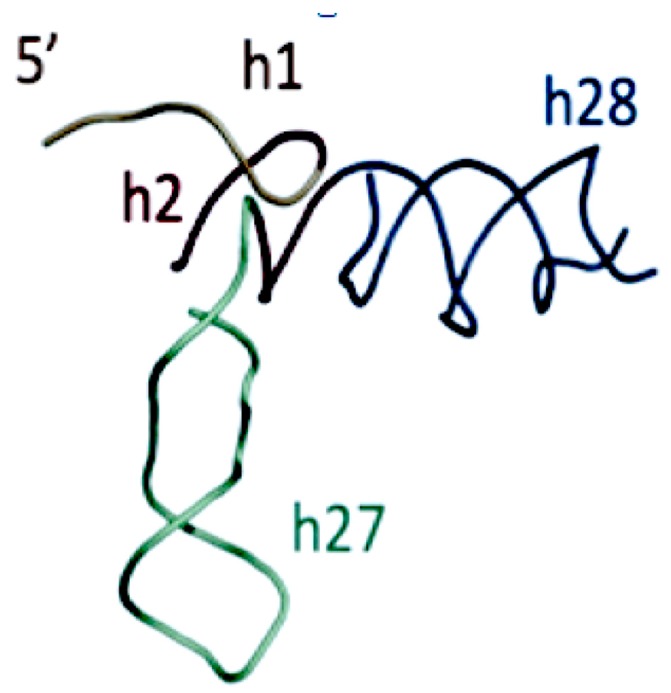
Proposed SSU seed, domain A Gulen et al. [37]. The SSU domain A was proposed to include SSU helices H27 and H28, resulting in a structure similar to the structure of the “boomerang” tRNA intermediate proposed structure in Figure 7C. Figure 9 has been provided by Anton Petrov.

Gulen [37] proposed, that the Domain A of the extant SSU plays a crucial role in the SSU by forming a scaffold linking the other SSU domains, Figure 9. As such, it was proposed as the starting center, or seed, for an accretion model of the SSU. There is an interaction in this substructure, which mimics the elbow of the tRNA. The structure of this proposed SSU seed also has interesting similarities to the boomerang structure in Figure 7C, of the proposed intermediate form of the earliest Proto-tRNAs above.

This proposed seed supports the link between the tRNA accretion model and that of the ribosome subunits [38]. The merger of the arm with the stem loops, completes the search for the origin of the GC code. It is in coevolution of the catalytic domain of the class II Aminoacyl-tRNA synthetases and the XCCA and the operational code in the arm of the tRNA.

### 4.7. What Was the Function of the Proto-SSU?

The extant SSU’s functions involve the binding of a messenger RNA and a tRNA whose anticodon stem loop reads the codon on the messenger RNA. This is an evolved form of what happened with the proto-messenger RNA and with the stem loops containing the GC anticodons for the triplets in a proto-messenger RNA. The proto-message was due to the ligation of triplets and provided an alternate method of forming coded short peptides of Glycine, Proline, and Alanine. Then, as proposed above, the anticodon stem loops also contained a CCA-3′ end with an activated amino acid.

It is here proposed that the proto-SSU was where a coded form of peptide synthesis took place before interacting with the LSU. The stem loops which were synthesized from doublets and triplets resulted in stem loops with a GC code in the five base paired helix of the stem and with a GC code in the seven-membered nucleotide loop. This allowed the XCCA on the stem to form an ester with an amino acid catalyzed by a catalytic module of a proto-class II aminoacyl-tRNA synthetase. The amino acid ester on the CCA of a stem loop of a proto-tRNA was coded for by a codon on the proto-messenger RNA. Moreover, the adjacent amino acid ester on the CCA of stem loop proto-tRNA formed a coded dipeptide and, if continued formed a longer coded peptide. The proto-ribosomal subunits (LSU and SSU) began with their key functions independently before merging to form the ribosome. This later interdependence is supported by the full ribosomal accretion models of the Georgia Tech group [33,38].

## 5. tRNA and Replication

The above accretion model for the tRNA is also a model for the replication of the proto message as well as for the earliest form of the ribosomal subunits. “The earliest catalyst RNA ligase spliced two RNA molecules by forming 3′–5′ phosphodiester bond between the 3′ hydroxyl of one RNA molecule and the 5′ phosphate of another RNA molecule. It is, therefore, relatively easy to use a polymerized set of primitive single-stranded triplets as a template for the polymerization of a complementary set of triplets, which are polymerized by a primitive RNA ligase. The same complementarity between the triplets or doublets in the loops between the stem anticodon loops are used in translation and it would be no accident that replication and translation were similar processes” [39]. Thus, the origin of the GC code was a tale of three peptide catalysts: The nucleotidyltransferases CCA enzyme, the class II catalytic domains, and the RNA ligase. We can continue to trace the evolution of the genetic code with the ribosomal proteins.

### Ribosomal Proteins and the Evolution of the Genetic Code

The ribosome is a complex molecular machine found within all living cells, that serves as the site of biological protein synthesis (translation). Ribosomes link amino acids together in the order specified by messenger RNA (mRNA) molecules. Ribosomes consist of two major components: The small ribosomal subunits, which read the messenger RNA; and the large subunits, which join amino acids to form a polypeptide chain. Each subunit comprises one or more ribosomal RNA (rRNA) molecules and a variety of ribosomal proteins (r-protein). The ribosomes and associated molecules (e.g., the aminoacyl-tRNA synthetases) are also known as the translational apparatus.

There has been a coevolution between the tRNA and the Ribosome. This has been observed at two different levels. First there has been an extensive accretion model developed for the evolution of the ribosome. In its early phases, it parallels that of the above tRNA accretion model for the tRNA [33]. Second, an analysis of the ribosomal proteins has provided insight as to a sequence in the emergence of different structural types of peptides and the resulting block structure of the ribosomal proteins. Most important here are the large subunit protein extensions to the PTC (peptidyl transferase center). These are largely without secondary structure and are rich in Alanine, Proline, Glycine, and positive amino acids, implying that they were early-encoded peptides based on a GC code. The other key characteristic of the ribosomal proteins is that they appear to have been assembled from multiple shorter peptides. This is reflected in their taxonomic division block structure [40]. Finally, the structural complexity of the ribosomal proteins increase as each one goes out from the random coils at the PTC to the tunnel, and finally to the surface of the ribosome, changing from simpler random coils, through mostly beta hairpins, to alpha beta domains and finally to alpha domains.

## 6. Conclusions

(1) The first replicators were self-replicating iron-rich clays which fixed carbon dioxide into oxalic and other dicarboxylic acids.

(2) “…this system of replicating clays and their metabolic phenotype, then evolved into the sulfide-rich region of the hot-spring acquiring the ability to fix nitrogen. The feedback between the clay surfaces and the early metabolic products, oxalic acid, citric acid amino acids, and short peptides result in the synthesis of the clays themselves. It is this feedback, which is involved in the selective forces, that expands the metabolic network. Finally, phosphate was incorporated into the evolving system, which allowed the synthesis of nucleotides and phospholipids. If biosynthesis recapitulates biopoiesis [41], then the synthesis of amino acids preceded the synthesis of the purine and pyrimidine bases. Furthermore, the polymerization of the amino acid thioesters into polypeptides preceded the directed polymerization of amino acid esters by polynucleotides. “Thus, the origin and evolution of the genetic code is a late development and records the takeover of the clay by RNA” [42].

(3) The proteins of the translational apparatus have a memory of their evolution and thus we can read that record.

(4) The arrival of RNA in the biosphere is best understood by the origin and evolution of the tRNA. The remaining problem is the evolution of the GC code to the GCA code and to the GCAU code, as exemplified in the proteins of the translation apparatus.

(5) The origin of the genetic code began with a GC code. The evolution of the genetic code from the GC code to the GCA code is based on the further coevolution of the aminoacyl-tRNA synthetases and the proto-tRNA. For example, the entry of Glutamic acid into the coding system means that the catalytic domain of the class I aaRS, the Rossmann fold, must be introduced and its evolution worked out. The appearance of the wobble is visible in the A column of the genetic code: Glu/Asp, Gln/Hist, Lys/Asn (e.g., Glu (GA pur)/Asp (GA pyr) share the GA* codon). The wobble is related to the introduction of the anticodon domains of the class I and II synthetases. The coevolution of the proto-tRNA evolution with the evolution of the anticodon domain likely explains the origin of the wobble. The further evolution of the 3D structure of the tRNA, especially the elbow, involves the continuing evolution of the LSU and SSU of the ribosome and the interaction of those two subunits. These will be dealt with in our next paper.

## Figures and Tables

**Table 1:** The standard genetic code table, re-ordered to place the Guanine–Cytosine coding block in the upper left corner, and using the standard abbreviations for the twenty amino acids.

	G	C	A	U	
**G**	Gly	Ala	Glu	Val	G
					A
					------
			Asp		C
					U
**C**	Arg	Pro	Gln	Leu	G
					A
					------
			His		C
					U
**A**	Arg	Thr	Lys	Met	G
					A
					------
	Ser		Asn	ILe	C
					U
**U**	Trp	Ser	Term	Leu	G
					A
	Term				------
	Cys		Tyr	Phe	C
					U
